# Comparison of human pluripotent stem cell differentiation protocols to generate neuroblastoma tumors

**DOI:** 10.1038/s41598-024-73947-y

**Published:** 2024-10-04

**Authors:** Bo Cheng, Wanqi Fang, Steven Pastor, Alexander R. March, Tania Porras, Hong-Wei Wu, Miriam Velez, Chintan Parekh, John M. Maris, Shahab Asgharzadeh, Miller Huang

**Affiliations:** 1 Cancer and Blood Disease Institutes, Children’s Hospital Los Angeles, The Saban Research Institute, 4650 Sunset Blvd #57, Los Angeles, CA 90027 USA; 2 Keck School of Medicine, University of Southern California, Los Angeles, CA USA; 3 Division of Oncology and Center for Childhood Cancer Research, Children’s Hospital of Philadelphia, Philadelphia, PA USA; 4 The Perelman School of Medicine at the University of Pennsylvania, Philadelphia, PA USA; 5 Department of Biomedical and Health Informatics, Childrens Hospital of Philadelphia, Philadelphia, PA USA

**Keywords:** Human pluripotent stem cells, Neuroblastoma, Sympathoadrenal cell, MYCN, Stem-cell differentiation, Cancer models, Cancer, Stem cells

## Abstract

Neuroblastoma is the most common pediatric extracranial solid tumor and is derived from trunk neural crest cells (tNCC) and its progenitor sympathoadrenal (SA) cells. While human pluripotent stem cell (PSC) models of neuroblastoma have been described, the PSC were differentiated using protocols that made neural crest cells, but not specifically the trunk subtype. Here, we compared four recent protocols to differentiate pluripotent stem cells (PSC) toward SA cells and examined their efficiency at generating SA cells along with earlier cell states (neuromesodermal progenitors [NMP], tNCC), as well as generating MYCN-driven tumors. Interestingly, the protocols that created cells with the highest level of NMP markers did not produce cells with the highest tNCC or SA cell markers. We identified a protocol that consistently produced cells with the highest level of SA markers using two PSC lines of different genders. This protocol also generated tumors with the highest level of PHOX2B, a marker of neuroblastoma. Transcriptionally, however, each protocol generates tumors that resemble neuroblastoma. Two of the protocols repeatedly produced adrenergic neuroblastoma whereas the other two protocols were ambiguous. Thus, we identified a protocol that reliably generates adrenergic neuroblastoma.

## Introduction

Neuroblastoma is a common childhood cancer that originates in tissues of the sympathetic nervous system, usually in the adrenal medulla or paraspinal ganglia, and can therefore appear as mass lesions in the neck, chest, abdomen, or pelvis^1,2^. The cell of origin of neuroblastoma is believed to be sympathoadrenal (SA) cells which initially were thought to give rise to sympathetic neurons of the paraspinal ganglia and chromaffin cells of the adrenal medulla^3,4^. Recent studies found a more complex trajectory where Schwann cell precursors from the neural crest can give rise to chromaffin cells and sympathoblasts can be derived from an immature chromaffin state^5–7^. SA cells are derived specifically from the trunk subtype of neural crest cells (tNCC) which are progenitors from neuromesodermal progenitors (NMP)^8,9^. Markers of SA cells have been found to be highly expressed in neuroblastoma tumors and constitute the core regulatory circuitry in the adrenergic subtype of neuroblastoma, including *PHOX2B*, *HAND2* and *GATA3*^10,11^. Indeed, the first genetically engineered mouse model of neuroblastoma was derived by mis-expression of the oncogene, *N-Myc*, in TH-expressing cells^12^. Similarly, zebrafish studies show that mis-expression of MYCN via the *DBH* promoter was sufficient to drive neuroblastoma formation^13^. These studies implicate SA cells as a cell of origin for neuroblastoma.

While animal models have been useful in studying neuroblastoma driven by one or two genes, it is difficult to model chromosome copy number variations (CNV), a significantly more frequent recurring event than single nucleotide variations in neuroblastoma^14,15^, as genes along human and animal chromosomes do not align. Thus, there is a need for human stem cell models of neuroblastoma which have been recently described. In the initial studies, human pluripotent stem cells (PSC) were differentiated toward neural crest cells, transduced with MYCN and implanted into mice^16,17^. Another study created a mouse-human chimera model in which human neural crest cells were implanted in utero in gastrulating mouse embryos^18^. However, the differentiation protocols cited by these studies lacked a posteriorizing factors such as retinoic acid or BMP and we have previously shown that the lack of posteriorizing factors results in NCCs that express the cranial-specific transcription factor ETS1^19^. Therefore, the early protocols were likely biased towards cranial NCCs which would not model neuroblastoma as accurately as trunk NCC.

Between 2016 and 2018, four protocols were described to generate trunk NCC and/or SA cells from human PSCs in which the addition of factors such as retinoic acid, BMP and sonic hedgehog (SHH) were used^8,19–21^. While there are similarities in the pathways that are inhibited or activated, each protocol varies by a number of factors including growing cells in suspension or adherent on different matrix proteins, the timing of activation or suppression of different pathways, supplements and the base media. Thus, while these protocols are designed to generate the same cell type, the resulting cells are likely expressing markers at different levels. It is also unclear how these protocols compare in generating NMPs, tNCC and SA cells. Furthermore, how would tumors differ when using different differentiation protocols of the same PSC? To identify an optimal protocol to generate tumors that best resemble neuroblastoma, here, we evaluate how each of these protocols compare in generating NMP, tNCC, SA cells and neuroblastoma tumors.

## Results

### Protocols #2 (Abu-Bonsrah 2017) and 3 (Frith, 2018) generate cells that best resemble NMP at day 3

**Fig. 1:**
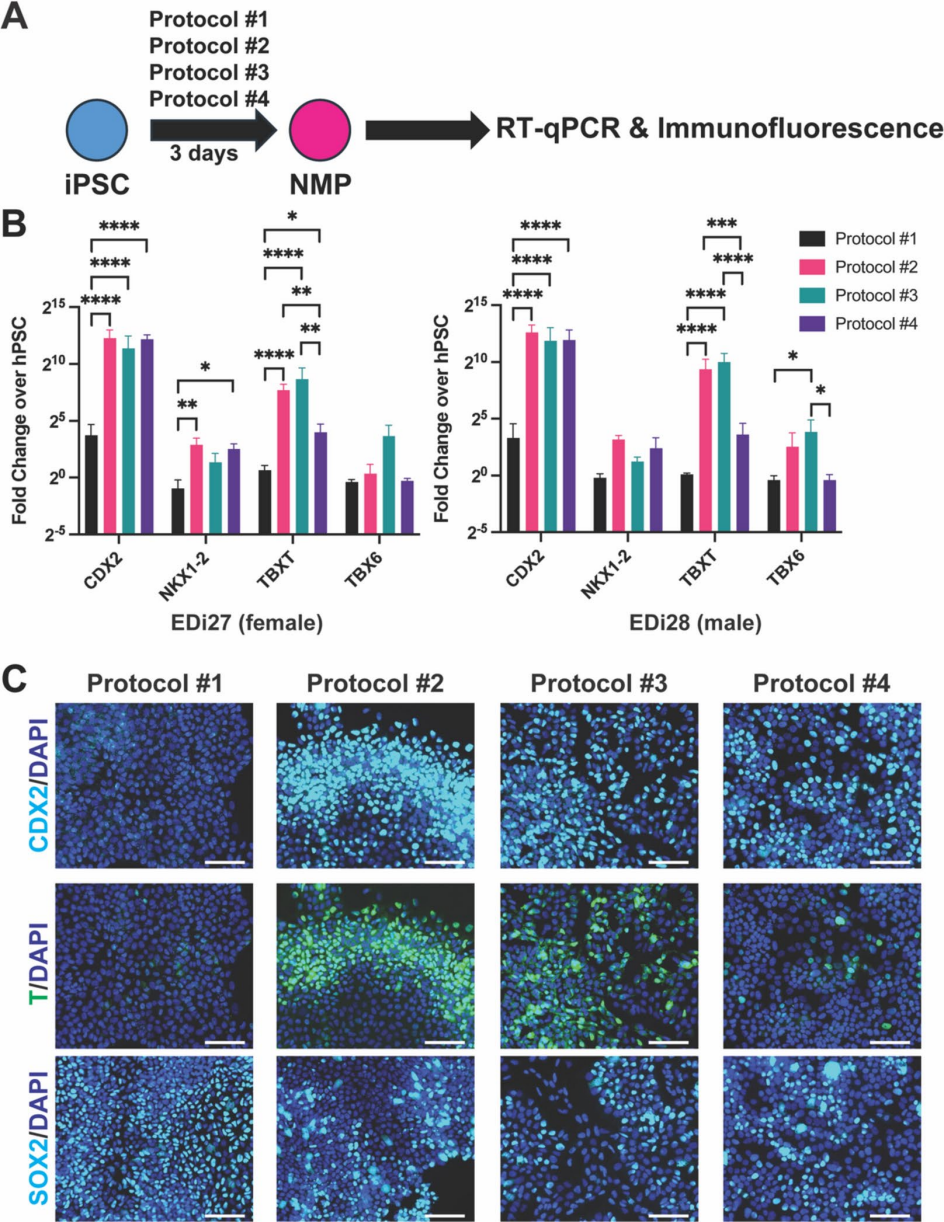
Protocols #2 and 3 generate cells with the highest NMP markers at day 3 of differentiation. (**A**) Schematic of analyzing cells differentiated from iPSC using four different protocols toward NMPs at day 3. (**B**) RT-qPCR analysis of NMP markers in *(left)* EDi27 and *(right)* EDi28 cells. n = 3, error bars represent standard error of mean. *p < 0.05, **p < 0.01, ***p < 0.001, ****p < 0.0001. (**C**) Immunofluorescence analysis of each protocol at day 3 for CDX2, T (Brachyury) and SOX2 co-stained with DAPI. Scale bar = 180 um.

To account for sex as a biological variable in the differentiation process, we used both a female (EDi27) and a male (EDi28) induced pluripotent stem cell (iPSC)^22^. While each protocol differs by a number of variables (e.g. base media components, extracellular matrix coating, adherence vs suspension), some notable differences for each protocol are prolonged treatment with retinoic acid (Protocol #1), activation of BMP signaling (BMP2, Protocol #2), moderate followed by high activation of BMP signaling (BMP4) and activation of SHH signaling (Protocol #3), retinoic acid and activation of BMP signaling (BMP4, Protocol #4). Two of the protocols we are testing described expression of NMP markers at day 3^8,21^. Therefore, we analyzed EDi27 and EDi28 cells after three days of differentiation using each of the four protocols for NMP markers TBX6, TBXT (also known as T or Brachyury), CDX2 and NKX1-2^23–27^ (Fig. S1, Fig. 1A). At the mRNA level, RT-qPCR analysis at day 3 showed that Protocols #2 and 3 had the highest level of marker expression for *CDX2*, *NKX1-2*, *TBXT* and *TBX6* (Fig. 1B). At the protein level, we performed immunofluorescence for CDX2, T (Brachyury) and SOX2. Protocols #2 and 3 showed positive staining for all three markers, whereas Protocol #4 was negative for Brachyury and Protocol #1 was negative for CDX2 and Brachyury (Fig. 1C, S2). To ensure the cells were exiting the pluripotent stage despite the positive expression of SOX2, we analyzed the expression of pluripotency markers *POU5F1* and *NANOG* by RT-qPCR. Expression of each marker was reduced compared to pluripotent stem cells indicating the cells have lost pluripotency (Fig. S3). Taken together, Protocols #2 and 3 generate cells with the highest expression of NMP markers at day 3 of differentiation.

### Protocol #4 (Kirino, 2018) generates cells that best resemble tNCC at day 8

**Fig. 2:**
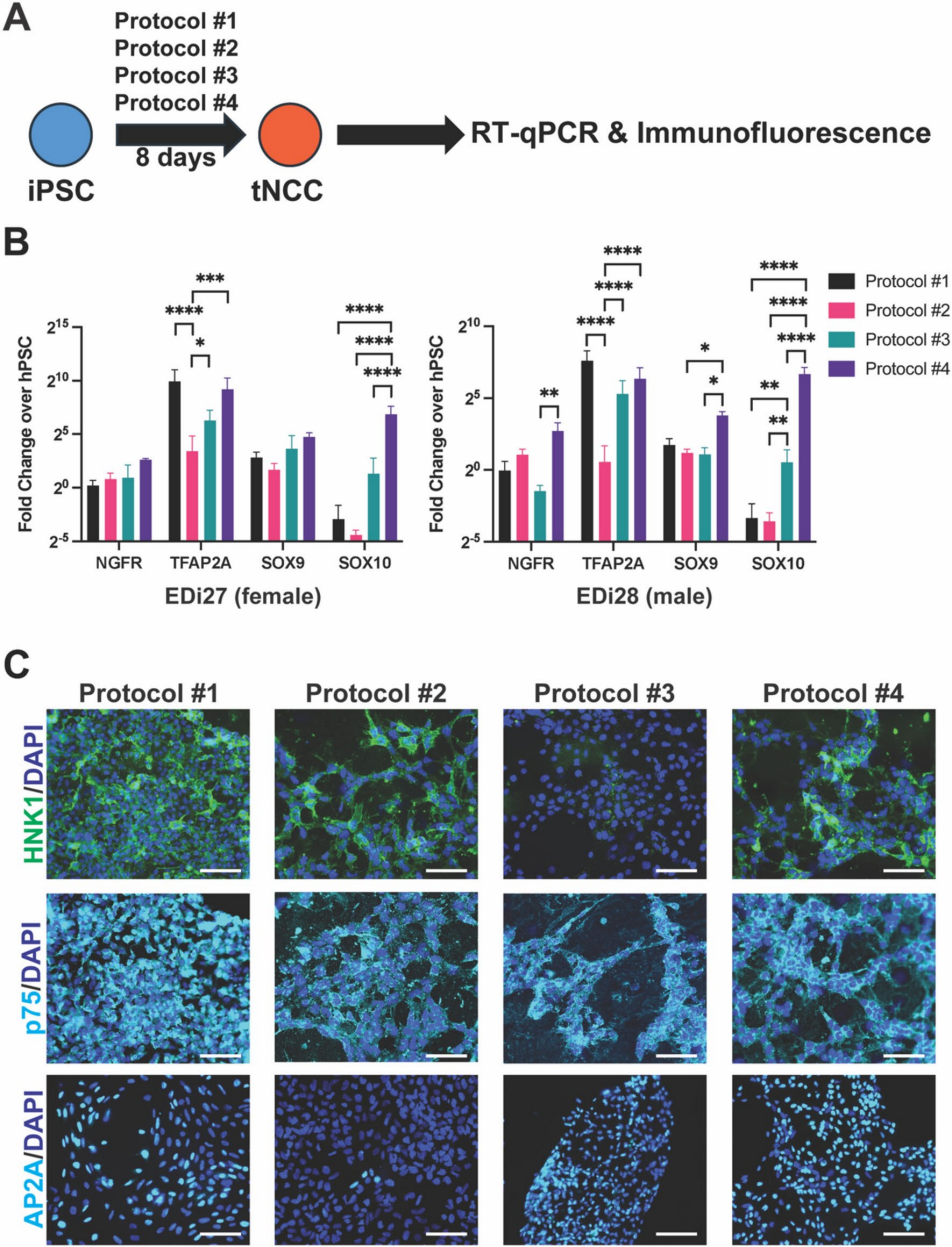
Protocol #4 generates cells that best resemble NCC at day 8 of differentiation. (**A**) Schematic of analyzing cells differentiated from iPSC using four different protocols toward tNCC at day 8. (**B**) RT-qPCR analysis of tNCC markers in *(left)* EDi27 and *(right)* EDi28 cells. n = 3, error bars represent standard error of mean. *p < 0.05, **p < 0.01, ***p < 0.001, ****p < 0.0001. (**C**) Immunofluorescence analysis of each protocol at day 8 for HNK1, p75, and AP2a co-stained with DAPI. Scale bar = 180um.

Next, we analyzed how each protocol generates tNCC. Since Protocol #3 changes from tNCC media to SA media at day 8 and the other protocols are using tNCC media at day 8, we chose to analyze tNCC markers at this time point (Fig. 2A). First, we analyzed pan-NCC markers *TFAP2A*, *NGFR, SOX9*, and *SOX10* by RT-qPCR. Protocol #4 showed high expression for all four genes, whereas Protocol #2 showed the lowest expression for each NCC marker (Fig. 2B). At the protein level, Protocol #4 showed positive staining for HNK1, p75 and AP2a while Protocol #1 showed stronger expression for each of these proteins (Fig. 2C, S4). Protocol #2 was positive for HNK1 and AP2a, but negative for p75. Protocol #3 was positive for p75 and AP2a, but negative for HNK1. Thus, while each Protocol can express NCC markers, Protocol #4 was the most consistent at showing high expression of each NCC marker tested.

To determine whether the NCCs generated are the trunk subtype, we examined expression of various HOX genes. Specifically, we looked at *HOXB4-9* and *HOXC8-9* which have been described to be expressed in tNCC^8,28–30^. RT-qPCR analysis showed no difference in expression of *HOXB4-7* among the four protocols (Fig. S5). For *HOXB8-9* and *HOXC8-9*, Protocol #1 had the lowest expression of each of these genes, whereas no difference was seen in cells produced by Protocols #2, 3 and 4 (Fig. S5). Altogether, Protocol #4 generated cells that best resemble tNCC at day 8 of differentiation.

### Protocol #4 (Kirino, 2018) generates cells that best resemble SA cells at day 12

**Fig. 3:**
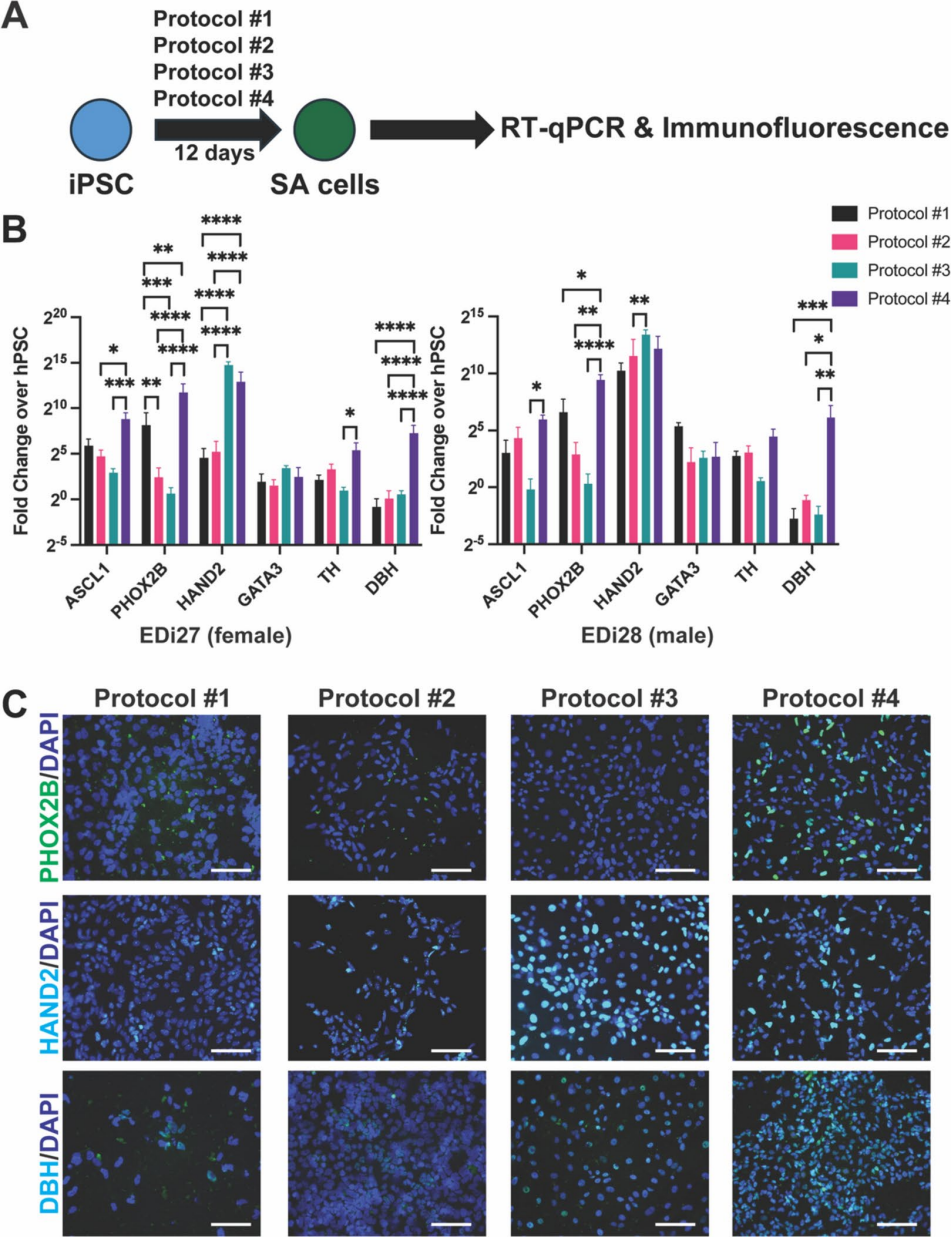
Protocol #4 generates cells that best resemble SA cells at day 12 of differentiation. (**A**) Schematic of analyzing cells differentiated from iPSC using four different protocols toward SA cells at day 12. (**B**) RT-qPCR analysis of tNCC markers in *(left)* EDi27 and *(right)* EDi28 cells. n = 3, error bars represent standard error of mean. *p < 0.05,**p < 0.01,***p < 0.001,****p < 0.0001. (**C**) Immunofluorescence analysis of each protocol at day 12 for PHOX2B, HAND2, and DBH co-stained with DAPI. Scale bar = 180 um.

We then evaluated how each protocol generated SA cells. We chose day 12 as the SA timepoint since Protocols #1 and 2 end by day 11 and Protocols #3 and 4 have cells grown in media to promote SA cell generation (Fig. 3A). We analyzed expression of SA markers *ASCL1, PHOX2B, HAND2, GATA3, TH* and *DBH*. RT-qPCR analysis of each protocol showed Protocol #4 cells expressing the highest level of *ASCL1, PHOX2B, TH* and *DBH* (Fig. 3B). Protocol #3- and 4-generated cells had the highest expression of *HAND2* in EDi27 cells, whereas all four Protocols had similar levels of *HAND2* in EDi28 cells (Fig. 3B). Expression of GATA3 was relatively similar amongst all four protocols. Next, we performed immunofluorescence for protein expression of PHOX2B, HAND2 and DBH. Protocol #4 showed positive staining for each of these markers, whereas Protocol #1 was negative or had low expression for HAND2 and DBH, Protocol #2 was negative or had low expression for each of the three SA markers, and Protocol #3 was negative for PHOX2B (Fig. 3C, Fig. S6). Thus, Protocol #4 appear to generate cells with the highest expression of SA markers at day 12 of differentiation.

### Protocols #3 (Frith, 2018) and 4 (Kirino, 2018) generate tumors that best resemble adrenergic neuroblastoma

**Fig. 4:**
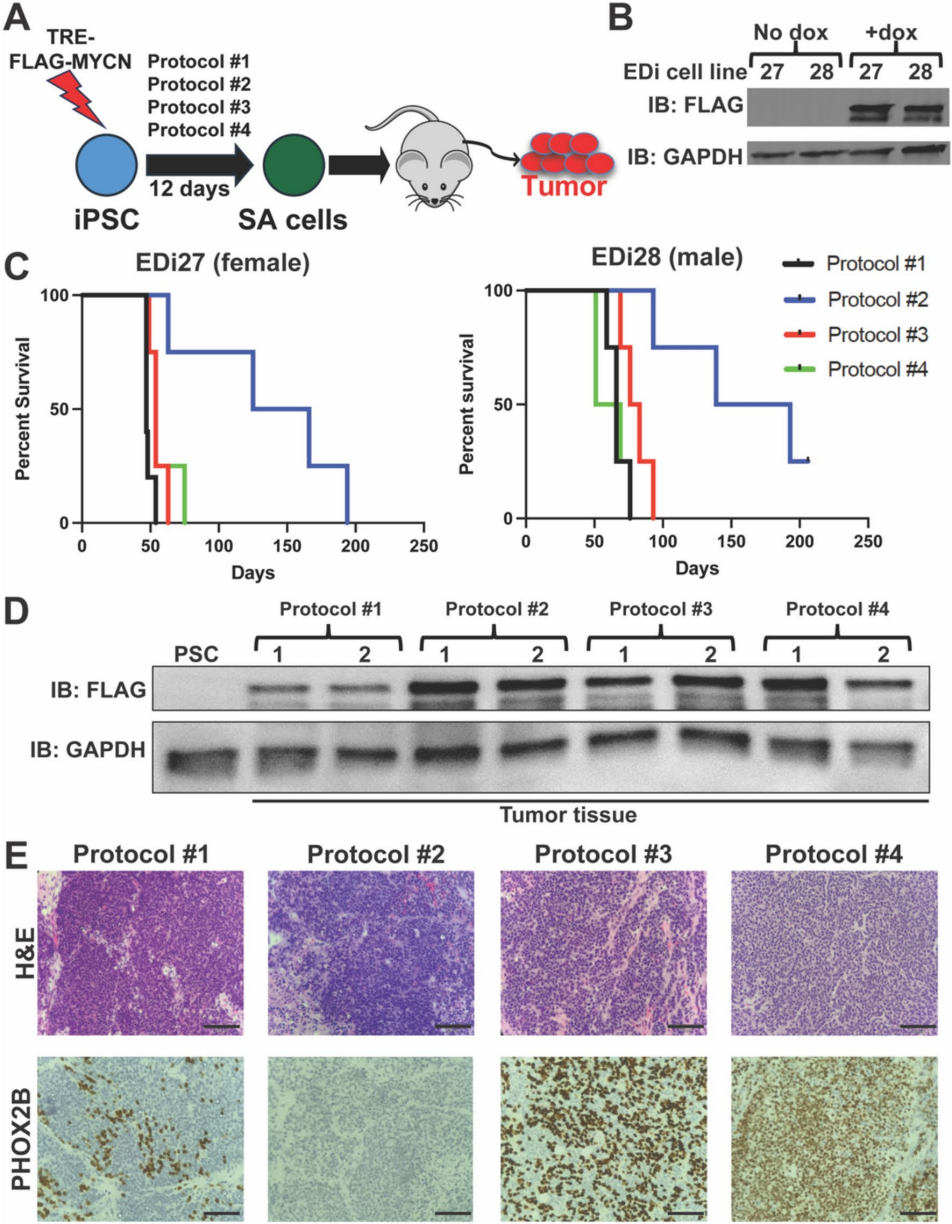
Protocol #4 generates tumors with the highest level of PHOX2B. (**A**) Schematic of generating tumors using each differentiation protocol in TRE-FLAG-MYCN transduced iPSC. (**B**) Western blot analysis of EDi27 and EDi28 TRE-FLAG-MYCN iPSC untreated and treated with doxycycline (100 ng/mL) for 24 h. (**C**) Kaplan Meier survival curves of mice implanted with *(left)* EDi27 (n = 4 per group) and *(right)* EDi28 (n = 4 per group) TRE-MYCN SA cells differentiated using each of the four protocols. (**D**) Western blot for expression of FLAG-MYCN in two tumors for each protocol compared to EDi27 iPSC. (**E**) Histology analysis of EDi27 MYCN tumors derived from each protocol showing *(top)* H&E staining and *(bottom)* IHC for PHOX2B. Scale bars = 100 um.

We next sought to determine which protocol would generate tumors that most resemble neuroblastoma. We and others have shown that MYCN is sufficient to transform NCC to tumors resembling neuroblastoma^16,17,31^. Therefore, we transduced EDi27 and EDi28 iPSC with doxycycline-inducible MYCN (TRE-MYCN) and differentiated using each of the four protocols until day 12 and injected into renal capsules of immunocompromised NSG mice which were fed on dox chow (Fig. 4A,B, Fig. S7). Protocols #1, 3, and 4 generated tumors at similar latencies ranging from days 47–75 with EDi27 and days 51–93 with EDi28 cells (Fig. 4C). Interestingly, Protocol #2 generated tumors with significantly longer latencies compared to the other protocols using both EDi27 (days 63–194) and EDi28 (days 93–193) (Fig. 4C). Expression of FLAG-MYCN in each EDi27 tumor was verified by western blot analysis (Fig. 4D, Fig. S7). Histological analysis of EDi27 tumors via H&E staining showed tumors from each protocol with small round blue cell morphology, similar to embryonal tumors including neuroblastoma (Fig. 4E). Interestingly, immunohistochemical staining for PHOX2B, a marker that distinguishes neuroblastoma amongst small round blue cell tumors^32^ was differentially expressed. PHOX2B was absent from tumors derived from Protocol #2, moderately expressed in tumors from Protocol #1, and highly expressed from either Protocol #3 or 4 (Fig. 4E, Fig. S8).

**Fig. 5:**
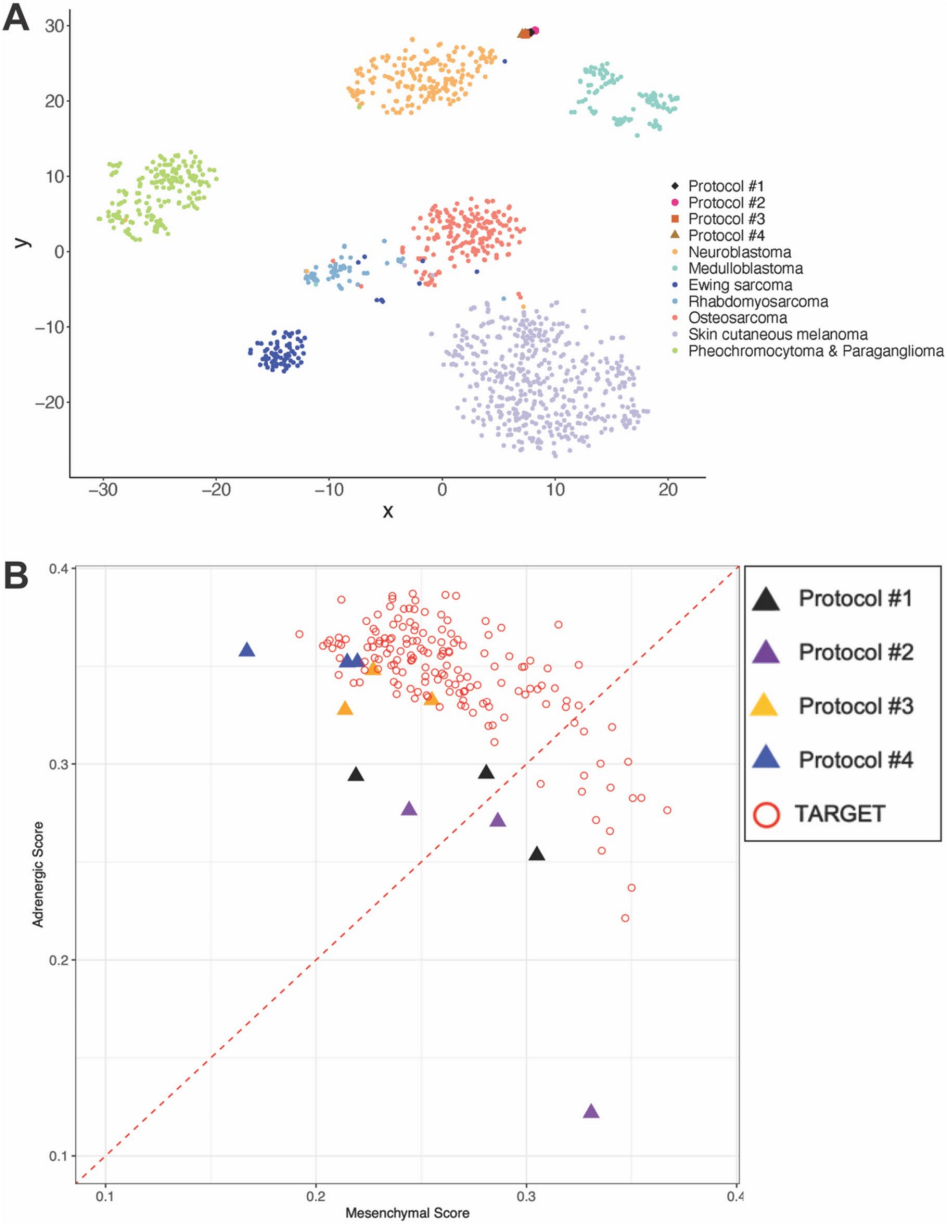
Protocol #3 and 4 generate adrenergic neuroblastoma tumors. (**A**) RNA was extracted from three EDi27 tumors from each protocol and analyzed by RNA-seq. Transcriptome profiles of human EDi27 tumors were compared against neuroblastoma, other human neural crest derived tumors (melanoma, pheochromocytoma and paraganglioma), and other small round blue cell tumors (medulloblastoma, ewing sarcoma, rhabdomyosarcoma). Patient data was obtained from the Treehouse Childhood Cancer Initiative (**B**) Transcriptomes of each EDi27 tumor was compared against neuroblastoma tumors based on the adrenergic score vs mesenchymal score. Neuroblastoma patient data was obtained from the TARGET database.

Next, we performed RNAseq on each EDi27 tumor to determine which tumor type each one resembled. We used tSNE analysis to compare the transcriptomes of our PSC-derived tumors with neuroblastoma and other neural crest derived tumors (pheochromocytoma & paraganglioma, melanoma) and other small round blue cell tumors (medulloblastoma, Ewing sarcoma, rhabdomyosarcoma). Surprisingly, in spite of the differences in cell type produced by each protocol, each PSC-derived tumor most closely resembled neuroblastoma (Fig. 5A). There was some separation amongst the four protocols which showed Protocols #3 and 4 were closer to neuroblastoma than Protocols #1 and 2 (Fig. 5A).

Since previous studies have shown that several neuroblastoma cell lines can exhibit features of an adrenergic or mesenchymal subtype, we asked whether the neuroblastoma tumors made by these protocols align more with an adrenergic or mesenchymal cell state. When compared to neuroblastoma tumors in the TARGET database, tumors from Protocols #3 and 4 closely resemble adrenergic neuroblastoma whereas the tumors from Protocols #1 and 2 were more heterogeneous where some tumors aligned with adrenergic subtype whereas others aligned with mesenchymal subtype (Fig. 5B). Thus, Protocols #3 and 4 were produced tumors that resembled adrenergic neuroblastoma.

### Stromal and immune cells contribute to molecular differences between human patient samples and PSC-derived tumors

**Fig. 6:**
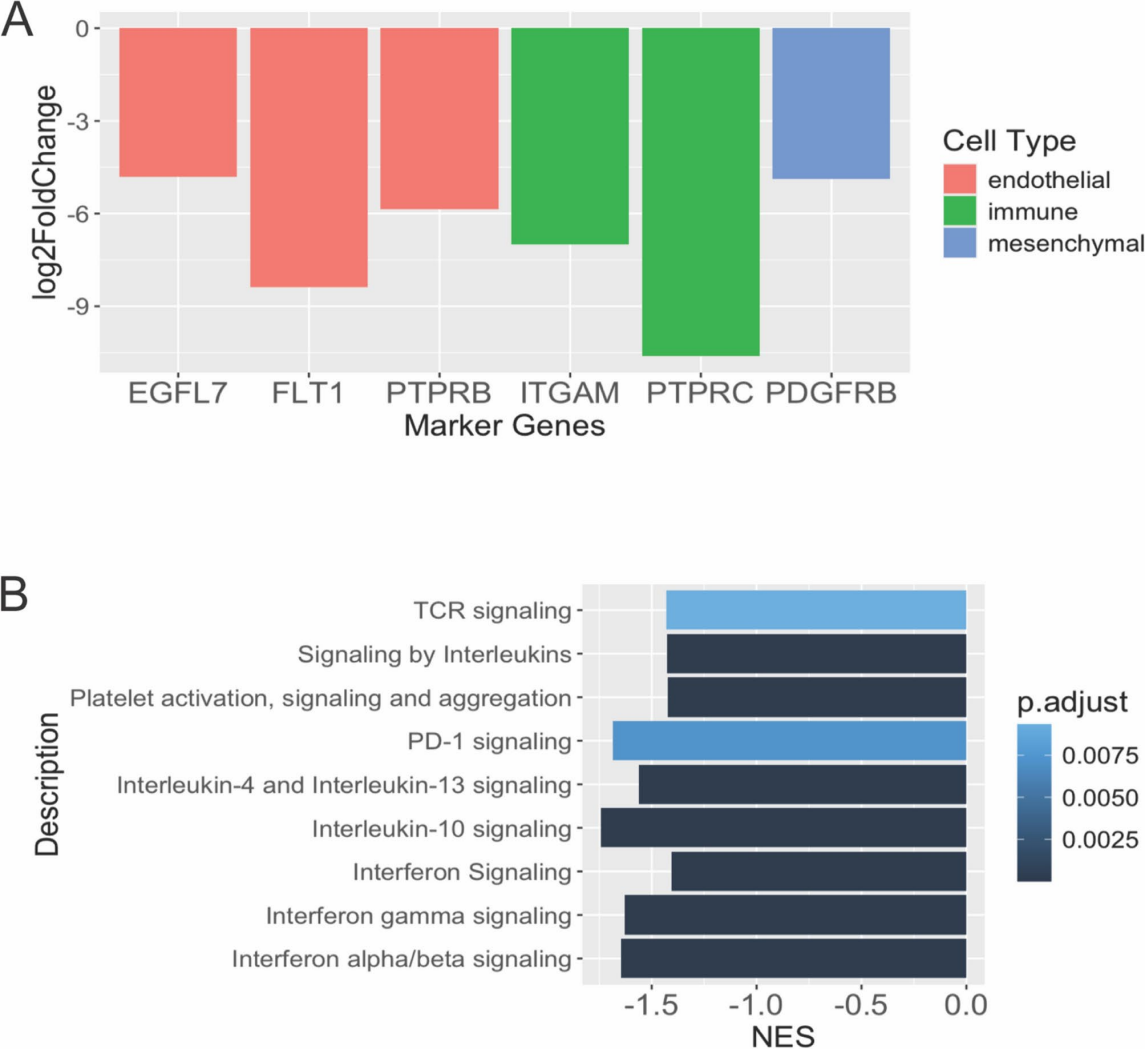
Stromal and immune cells contribute to molecular differences between human neuroblastoma patient samples and Protocol #4 tumors. (**A**) Expression of stromal and immune markers were analyzed between TARGET human *MYCN*-amplified neuroblastoma samples (> = 90% tumor purity, n = 6) and tumors from Protocol #4 (n = 3). Bars represent the log_2_ fold-change expression levels of the Protocol #4 tumors compared to the TARGET samples. (**B**) Gene ontology analysis of genes differentially expressed between Protocol #4 tumors and TARGET tumors using the REACTOME database.

Next, we examined whether remaining transcriptomic differences between human neuroblastoma patient samples and our PSC-derived tumors is due to the presence of stromal and immune cells in human patient samples. We analyzed the expression of endothelial markers (*EGFL7, FLT1, PTPRB*^33^), mesenchymal cells (*COL1A2, COL1A1, PDGFRB*^34,35^) and immune markers (*ITGAM, CD247, PTPRC*^36–38^) in *MYCN*-amplified neuroblastoma patient samples (with at least 90% tumor purity) and tumors from Protocol #4. Indeed, even though the patient samples had high purity, they still expressed higher level of several of these stromal and immune genes compared to the PSC-derived tumors (Fig. 6A, Supplementary Table 1). In support of this, we also performed gene ontology analysis of the transcriptomes and found a number of immune-related pathways (e.g. Toll-like receptor, TNF signaling, IL-17 signaling) were enriched in the patient samples compared to the PSC-derived tumors (Fig. 6B). Thus, stromal and immune markers contribute to the differences seen between human patient tumors and our PSC-derived tumors.

## Discussion

Human stem cell models are becoming a valuable system for cancer research that can faithfully recapitulate disease and are highly tractable. A major limitation to the human PSC-based models, however, is the variability in methods to generate the cell of origin. In some cases, a specific cell type is difficult to identify due to lack of defined set of cell markers and it is also unclear the level of expression each marker needs to be to properly represent that cell type. How these differences could influence a tumor type is also unknown. Here, we tested four different protocols that are used to generate tNCC and SA cells to evaluate how different protocols can influence producing MYCN-driven neuroblastoma.

Interestingly, the expression of markers at earlier cell states did not necessarily translate to similar expression levels of later cell states. For instance, while Protocol #2 produced high levels of NMP markers at day 3, it generated cells with some of the lowest level of tNCC and SA markers at later time points. Although Protocol #1 produced cells with the lowest expression of NMP markers at day 3, these cells expressed the high level of NCC markers at day 8 (but also the lowest level of tNCC-specific HOX genes). Thus, optimization of cell marker expression at intermediate cell states may not produce cells with the highest expression of markers in the final cell state.

We also sought to determine how each differentiation protocol influenced the phenotype of tumors that ultimately arose. In both EDi27 and EDi28 cell lines, Protocols #1–3 produced tumors at similar latency and penetrance, whereas Protocol #2 had a significantly longer latency. Histologically, expression of the neuroblastoma marker PHOX2B was variable across each protocol with Protocol #4 derived tumors expressing the most PHOX2B. In spite of these differences amongst each tumor, the global transcriptome of each tumor did not show many differences and each tumor resembled neuroblastoma when compared to other neural crest derived tumors and small round blue cell tumors. While neuroblastoma tumors from Protocols #3 and 4 were clearly more adrenergic than mesenchymal, the subtype was more ambiguous with tumors derived from Protocols #1 and 2. Since mesenchymal neuroblastoma has been linked to a more undifferentiated neural crest cell state than the adrenergic subtype, the subtype differences in our tumors could be a reflection of Protocols #1 and 2 at day 12 resembling tNCC more than SA cells, whereas Protocols #3 and 4 at day 12 are more firmly in the SA cell states. Therefore, we found each protocol can generate tumors that resemble neuroblastoma at the global transcriptomic level but differ in other features such as latency, expression of PHOX2B and subtype of neuroblastoma. Based on our findings, Protocol #4 is the most reliable at generating SA cells and neuroblastoma tumors with expression of PHOX2B.

One caveat to our findings is after we began this project, additional protocols were described to generate tNCC via high expression of the WNT activator, CHIR99021 (10uM vs the 2uM used in Protocol #4)^39,40^. It would be of interest to determine how these protocols would compare with Protocol #4 in generating SA cells and neuroblastoma tumors. Additionally, while the elevated PDGFB and TGFB signaling in patient samples is likely due to the stromal and immune cells (Fig. 6)^41^, it is possible activation of these signaling pathways could improve the efficiency of generating SA cells and neuroblastoma tumors. Lastly, while Protocol #4 may be optimal to MYCN-driven neuroblastoma, a non-MYCN-driven neuroblastoma may be better represented by a different protocol and require optimization.

One challenging aspect of the human stem cell model of neuroblastoma is the inability to generate a stable SA cell or tNCC population that can self-renew. To analyze different genotypes in a human stem cell neuroblastoma model, we need to constantly rederive tNCC or SA cells from the PSC state. In addition, if a genetic alteration influences differentiation from PSC to tNCC or SA cells (e.g. MYCN^16,17,31^), the gene needs to be regulated in an inducible way, such as a doxycycline-inducible promoter. In contrast, neuroepithelial stem (NES) cells are a stable cell population which we successfully used to analyze the effect of multiple genotypes to generate medulloblastoma tumors^42^. Since NES cells are a stable population, we are able to generate transgenic stable NES cell lines without the concern of blocking differentiation from the PSC cell state. Thus, identifying conditions that would allow for self-renewal of tNCC or SA cells would significantly improve the ease of using the human stem cell model of neuroblastoma to analyze multiple genotypes.

In conclusion, we provide data suggesting the protocol published by Kirino et al.^21^ can reliably be used to generate adrenergic neuroblastoma. These models will be essential to identify novel genetic drivers of the disease, and in particular, address the role of chromosome copy number changes in neuroblastoma (a significantly more frequent event than single nucleotide variations^14,15^) as genes on mouse and human chromosomes do not align.

## Methods

### Animals

Immunocompromised (NOD-scid IL2Rgamma^null^ or NSG) 6–8 -week old female mice used for transplantation were purchased from Jackson Labs. Mice were maintained in the Animal Care Facility at CHLA. All experiments were performed in accordance with national guidelines and regulations, and with the approval of the Institutional Animal Care and Use Committee at CHLA. For each in vivo experiment, 10^6^ NCC were injected into the left renal capsules of the mice. Mice were euthanized at endpoint, which was determined by one of two criteria: (1) either 2 cm length of tumor, or (2) 1 year post transplantation. Mice were euthanized by CO2 followed by cervical dislocation. Mice were fed doxycycline grain-based rodent diet (Bio-Serv) immediately following surgical recovery. The study is reported in accordance with ARRIVE guidelines.

### iPSC culture

Male EDi28 (Cedars Sinai Biomanufacturing Center) of passages 18–30 and Female EDi27 iPSC (Cedars Sinai Biomanufacturing Center)^22^ of passages 23–35 were maintained on GelTrex coated 6-well plates in StemFlex media (Thermo Fisher) in a humidified 37 °C incubator with 5% CO_2_. Cells were passaged every 4–5 days using Versene (Thermo Fisher) when approximately 80% confluent. iPSC were frozen using Stemcell Banker (Amsbio). All experiments using iPSC in the Huang lab were approved by the CHLA Stem Cell Research Oversight Committee. Cell lines were validated by STR analysis and mycoplasma tested every 2–4 weeks using Plasmotest (Invivogen).

### Differentiation of iPSC to SA cells

Differentiation from iPSC to tNCC was performed as previously described for each protocol^8,19–21^: 

Protocol #1 (Huang, 2016): Base media consisted of DMEM/F-12 + Glutamax (Thermo Fisher), 2% Probumin BSA (Millipore), 1 × Penicillin Streptomycin (Thermo Fisher), 1 × Trace Elements A, B, C (Corning), 1 × NEAA (Thermo Fisher), 50 uM β-mercaptoethanol (Thermo Fisher), 50ug/mL ( +)-sodium L-ascorbate (Sigma Aldrich), 10ug/mL Holo Bovine Transferrin (Thermo Fisher), 200 ng/mL LONGR3 IGF1 (Sigma Aldrich), 10 ng/mL Heregulin β-1 (Peprotech), 10 ng/mL FGF2 (Peprotech). On Day 0 cells were plated on GelTrex (Thermo Fisher) at density of 92,000 cells/cm^2^ in the Base media with 2uM Thiazovivin. On Day 1, media was changed with Base media supplemented with 2uM Thiazovivin (Stemcell Technologies), 10uM SB431542 (StemRD), 3uM CHIR99021 (R&D Systems). On Day 2, media was changed with Base media supplemented with 10uM SB431542 and 3uM CHIR99021. On Days 3–12 Media was changed daily with Base media supplemented with 10uM SB431542, 3uM CHIR99021 and 10uM Retinoic Acid (Sigma Aldrich) and cells were passaged with Accutase (Innovative Cell Technologies) every 2–3 days and plated on GelTrex.

Protocol #2 (Abu-Bonsrah, 2017): Base media consisted of 1:1 ratio of Neurobasal-A (Thermo Fisher) and DMEM/F-12 + Glutamax supplemented with 0.5 × B27 without vitamin A (Thermo Fisher), 1 × N2 supplement (Thermo Fisher), 1 × ITS-A (Thermo Fisher), 0.3% Glucose (Thermo Fisher), 0.5 × Glutamax (to supplement Neurobasal-A, Thermo Fisher), 1 × Penicillin Streptomycin. On Day 0, cells were seeded at a density of 10,000 cells/cm^2^ in StemFlex media with 2uM Thiazovivin in 10ug/mL laminin (Thermo Fisher) coated plates. On Days 1–3, media was changed every other day to Base media supplemented with 10uM SB431542 and 3uM CHIR99021. On Days 5–12, cells were passaged with Versene (Thermo Fisher) at a 1:2 ratio into a similarly sized ultra-low attachment plate (Corning) in Base media supplemented with 10 ng/mL BMP2 (Peprotech) and 20 ng/mL FGF2. Media was changed every other day.

Protocol #3 (Frith, 2018): Day 0–2 media consisted of 1:1 ratio of Neurobasal-A and DMEM/F-12 + Glutamax supplemented with 1 × B27 without vitamin A, 1 × N2 supplement, 1 × NEAA, 0.5 × Glutamax (to supplement Neurobasal-A), 1 × Penicillin Streptomycin, 100uM β-mercaptoethanol, 3uM CHIR99021, 20 ng/mL FGF2. On Day 0 cells were plated at density of 55,000 cells/cm2 in 10ug/mL vitronectin (Thermo Fisher) coated plates in Day 0–2 media supplemented with 2uM Thiazovivin. Media was changed on Day 1 without Thiazovivin. Day 3–7 media consisted of DMEM/F-12 + Glutamax supplemented with 1 × N2 supplement, 1 × NEAA, 2uM SB431542, 1uM CHIR99021, 1uM DMH-1 (R&D systems), 15 ng/mL BMP4 (Thermo Fisher). Cells were dissociated with Accutase on Day 3 and plated at a density of 30,000 cells/cm^2^ on GelTrex in Day 3–7 media supplemented with 2uM Thiazovivin. Media was changed on Days 5 and 7. Day 8–12 media consisted of Brainphys media (Stemcell Technologies) supplemented with 1 × B27 without vitamin A, 1 × N2 supplement, 1 × NEAA, 1 × Glutamax, 50 ng/mL SHH C24II (Genscript), 50 ng/mL BMP4 (Thermo Fisher), 1.5uM Purmorphamine (Sigma Aldrich). Cells were dissociated with Accutase on Day 8 and plated at a density of 20,000 cells/cm2 in Day 8–12 media supplemented with 2uM Thiazovivin. Media was changed daily.

Protocol #4 (Kirino, 2018). Day 0–2 media consisted of E6 media (Thermo Fisher) supplemented with 10uM SB431542, 2uM CHIR99021. On Day 0 cells were seeded at 300,000 cells/well in a 6-well ultra-low attachment plate supplemented with 2 mL of Day 0–2 media and 2uM Thiazovivin. On Day 1, 1 mL of additional media was added to the existing 2 mL of conditioned media. Day 3–9 media consisted of E6 media supplemented with 20 ng/mL FGF2, 100 nM Retinoic Acid, 50 ng/mL BMP4 (R&D systems). Media was changed every other day starting on Day 3. Day 10–12 media consisted of Neurobasal-A media supplemented with 1 × Glutamax, 1 × N2 supplement, 1 × B27 supplement without vitamin A, 20 ng/mL FGF2, 20 ng/mL EGF (Peprotech), 50 ng/mL BMP4 (R&D systems), 2ug/mL Heparin (Sigma Aldrich). Media was changed on Day 10.

### Generation of TRE-MYCN iPSC

EDi27 and EDi28 iPSC were transduced with lentivirus encoding TRE-MYCN as previously described^31^. Cells were selected using Blasticidin (10ug/mL) and treated with doxycycline for 24 h prior to FACS sorting for mScarlet signal.

### Antibodies

Primary antibodies for immunofluorescence were obtained commercially for CDX2 (1:200, Abcam), TBXT (1:200, R&D systems), SOX2 (1:100, Thermo Fisher), SOX9 (1:200, Cell Signaling Technology), HNK1 (1:250, Sigma Aldrich), p75 (1:50, Thermo Fisher), AP2A (1:100, Thermo Fisher), PHOX2B (1:50, Santa Cruz), HAND2 (1:100, Abcam), DBH (1:50, Santa Cruz). Secondary antibodies (1:500) were in the Alexa Fluor spectrum 488 and 647 (Thermo Fisher). Primary antibody for immunohistochemistry for PHOX2B (1:100) was from Abcam. Primary antibodies for western blot were also obtained commercially for FLAG (1:500, Sigma Aldrich) and GAPDH (1:10,000, GeneTex). Secondary HRP antibodies (1:5000) for western blot were obtained from BioRad.

### RT-qPCR

RNA was extracted using a Quick-RNA Miniprep kit (Zymo Research). 500 ng of RNA was converted to cDNA using VILO Superscript (Thermo Fisher) or the high-capacity cDNA Reverse Transcription Kit (Applied Biosystems) in a 20uL final volume and the following settings: 25 °C for 10 min, 42 °C for 60 min and 85 °C for 5 min. cDNA was then diluted in 80uL of water and qPCR was performed in a 384 well plate using SYBR green (Biorad) on an CFX384 machine (Biorad) with the following settings: 95 °C for 1 min, 40 cycles (95 °C for 3 s and 60 °C for 1 min). Each qPCR reaction occurred in final volume of 10uL containing 5uL SYBR mastermix, 5uM of each forward and reverse primer, and 0.4uL of the cDNA. Gene expression was normalized to GAPDH expression and represented as fold increase over the control cell lines.

### Immunohistochemistry

Extracted tumors from mice were fixed with 10% neutral buffered formalin (Sigma) for 24 h and subsequently stored in 70% ethanol (Sigma). Tumors were paraffin-embedded, sectioned, and stained for H&E and PHOX2B by the Histology Core at CHLA. Images were taken using the Revolve microscope (ECHO).

### Immunofluorescence

Cells were plated on appropriately coated cover slips (VWR) or Nunc Lab-Tek Chamber Slides (Thermo Fisher). Cells were fixed in 4% formaldehyde solution (Sigma-Aldrich), permeabilized with 0.3% Triton X-100 (Sigma) in 1 × DPBS (Thermo Fisher), and blocked for 1 h at room temperature with 1% BSA (Sigma) in 1 × DPBS. Coverslips were then incubated overnight at 4 °C with primary antibodies diluted in 1% BSA in 1 × DPBS, washed 3 times in 1 × DPBS, and incubated with secondary antibodies at room temperature for 1 h diluted in 1 × DPBS. Coverslips are mounted with Fluoromount-G mounting medium with DAPI (Thermo Fisher) on slides (Corning). Fluorescent images were taken using an Echo Revolve at 20x.

### Quantitation of immunofluorescence

Images were analyzed by ImageJ. After threshold adjustment, cells were segmented using the Binary Watershed tool, and quantified using the Analyze Particles tool. Expression of all markers were quantified over DAPI + cells in triplicate. Each percentage of marker-positive cells were calculated and represented as one data point in the plot. Statistical analysis was performed using GraphPad Prism (GraphPad).

### RNA-seq sample collection

Total RNA was extracted from flash frozen tissue using the Tissuelyser II (Qiagen) and AllPrep DNA/RNA Mini Kit (Qiagen). Quality of total RNA samples was checked on an Agilent Bioanalyzer 2100 RNA Nano chip (Agilent) and sent to Novogene (https://en.novogene.com/) for library preparation (polyA enrichment) and RNA sequencing (150 base pair Paired End reads, 50 million reads total).

### RNA sequencing and analysis

RNA sequencing reads were mapped to the human hg38 primary assembly reference genome using the HISAT2 aligner v2.2.0^43,44^. Gene expression was quantified as reads per Gene using featureCounts v2.0.2^45,46^ and the Ensembl release-109 Homo_Sapien gene annotations. T-distributed Stochastic Neighbor Embedding (t-SNE) was performed using the Rtsne R package^47^. Differential Gene expression analysis was performed using the DESeq2 R package^48^. Gene set enrichment was determined using the clusterProfiler R package^49–51^. Stromal cell content was determined by using previously defined canonical markers for immune, endothelial, and mesenchymal cells in differential expression analysis^33^. For this, models were compared to publicly-available TARGET MYCN-amplified samples and LFC values determined stromal cell differences^52^. Single sample scoring of adrenergic and mesenchymal molecular phenotypes were calculated using the singscore R package^53^ and the adrenergic and mesenchymal gene signatures described by van Groningen et al.^54^.

### Datasets

RNAseq data on MYCN-driven EDi27 SA tumors from each protocol are available in the Gene Expression Omnibus (GEO) database (GSE264211). Bulk RNA-seq data from the Treehouse Childhood Cancer Initiative (Tumor Compendium v11 Public PolyA (April 2020)) was obtained from https://treehousegenomics.soe.ucsc.edu/public-data/. Bulk RNA-seq data of the TARGET neuroblastoma cohort was obtained from https://portal.gdc.cancer.gov/projects/TARGET-NBL.

### Statistical analysis

For RT-qPCR, data points represent the average of 3 independent experiments ± standard error of mean and p values were generated by Two-Way ANOVA using GraphPad Prism version 10.3.1 for Mac, GraphPad Software, Boston, Massachusetts USA, www.graphpad.com. Statistical significance was interpreted as a p-value of less than 0.05. p values are represented as follows: ***p < 0.001, **p < 0.01, *p < 0.05, and not statistically significant (n.s.) when p > 0.05.

## Supplementary Information

Supplementary Information.

Supplementary Table 1.

## Data Availability

RNAseq data on MYCN-driven EDi27 SA tumors from each protocol are available in the Gene Expression Omnibus (GEO) database (GSE264211). Bulk RNA-seq data from the Treehouse Childhood Cancer Initiative (Tumor Compendium v11 Public PolyA (April 2020)) was obtained from https://treehousegenomics.soe.ucsc.edu/public-data/. Bulk RNA-seq data of the TARGET neuroblastoma cohort was obtained from https://portal.gdc.cancer.gov/projects/TARGET-NBL.

## Abbreviations

tNCC: Trunk neural crest cells
SA: Sympathoadrenal
iPSC: Induced pluripotent stem cells
