# Supplementary material for: Comparison of human pluripotent stem cell differentiation protocols to generate neuroblastoma tumors
Source: Sci Rep. 2024 Oct 4;14:23050. doi: 10.1038/s41598-024-73947-y (PMC11452544; doi:10.1038/s41598-024-73947-y)
Supplement: Supplementary file 1 — Supplementary Information. [file 41598_2024_73947_MOESM1_ESM.docx]

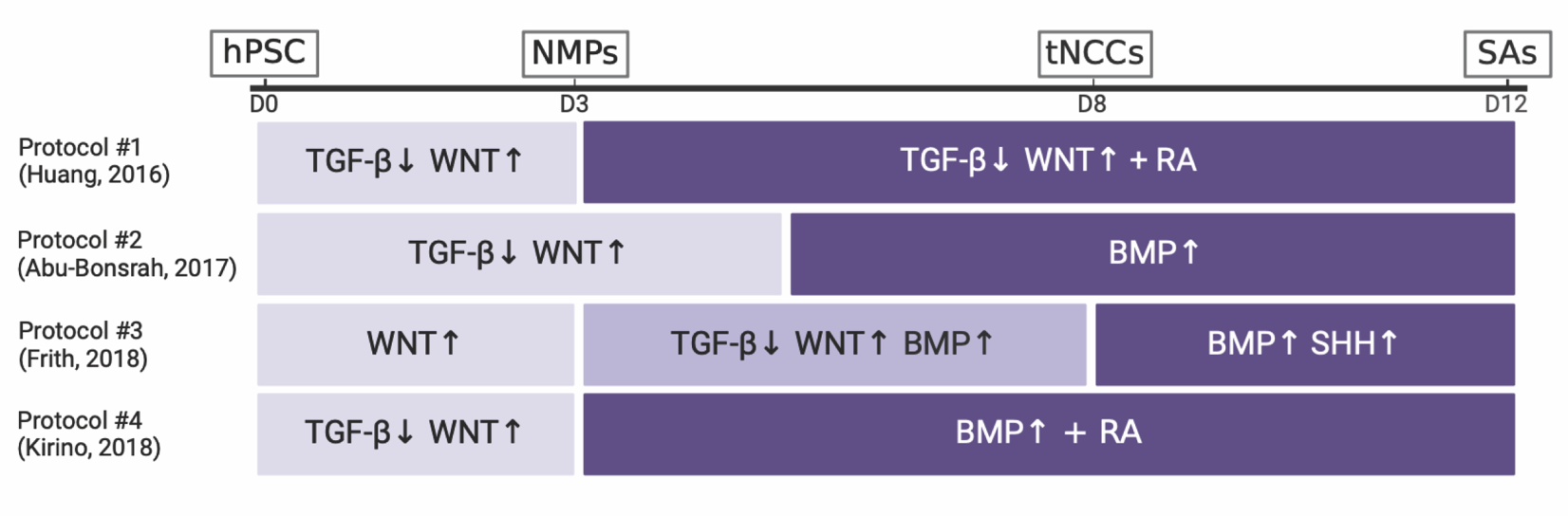


**Supplementary Figure 1: Overview of four differentiation protocols.** Schematic and timeline of the four differentiation protocols and the signaling pathways that are regulated at each time point. Color changes represent time points where media components change. The approximate cell state changes are day 3 for NMP, day 8 for tNCC and day 12 for SA cells.


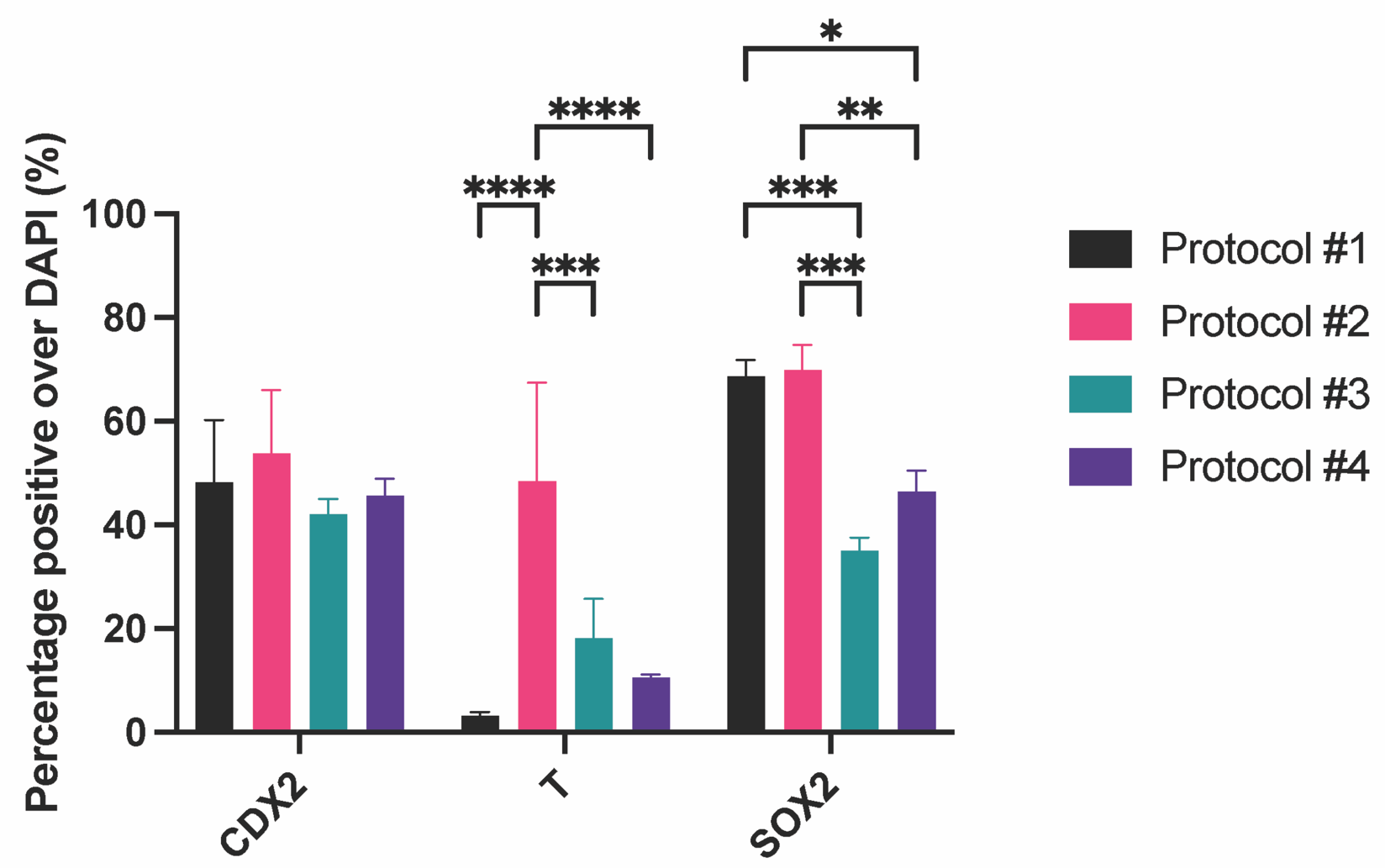


**Supplementary Figure 2: Quantitation of immunofluorescence for NMP markers.** Bar graph shows the percentage of number of positive cells for each protein compared to DAPI stained cells. n=3, error bars represent standard error of mean.


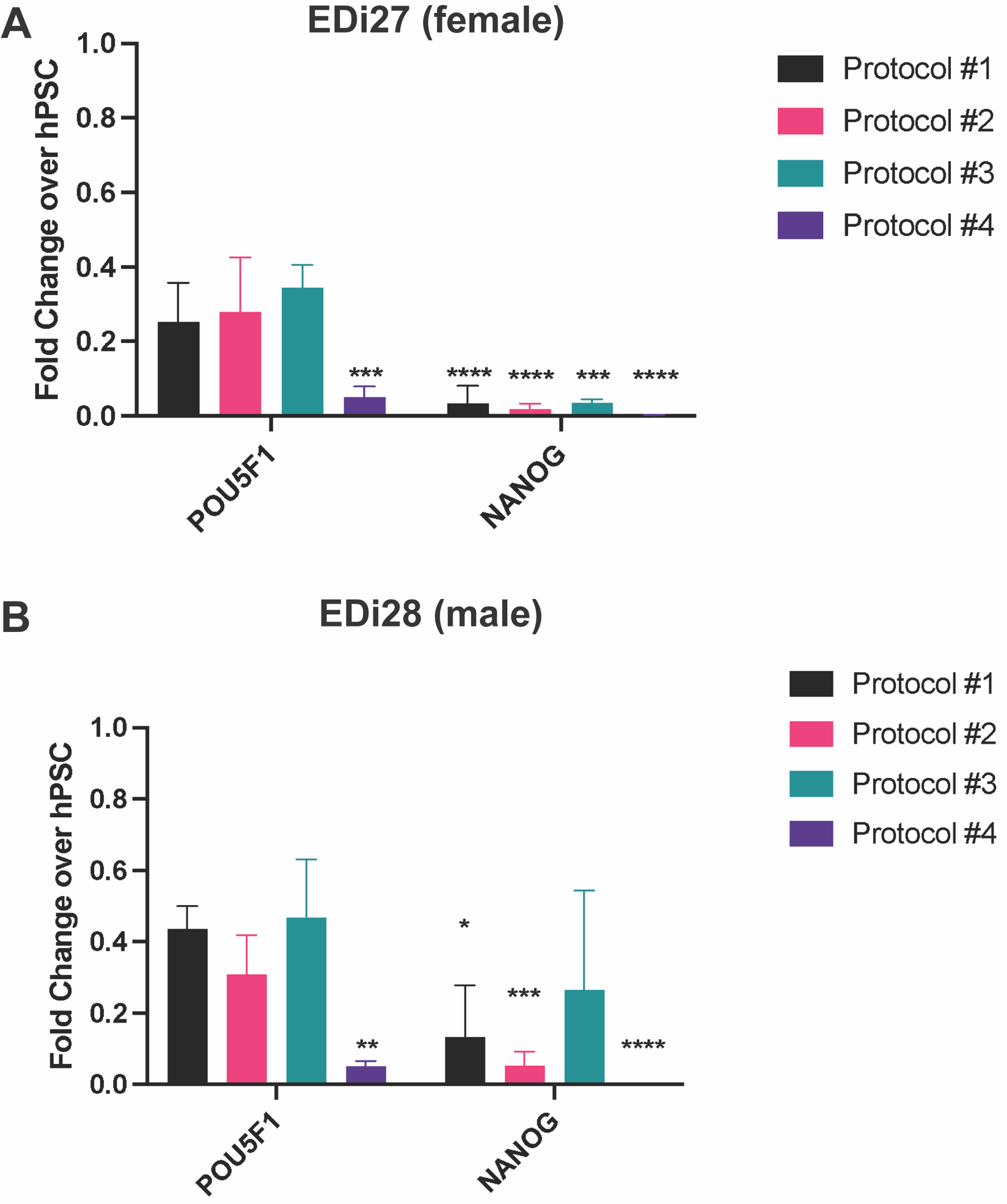


**Supplementary Figure 3: Expression of pluripotency markers are decreased by day 3 of differentiation. (A-B)** Bar graphs show expression of pluripotent markers *POU5F1* and *NANOG* are lower in each of the four protocols compared to hPSC in both EDi27 and EDi28 cell lines. n=3, error bars represent standard error of mean. *p<0.05,**p<0.01,***p<0.001,****p<0.0001


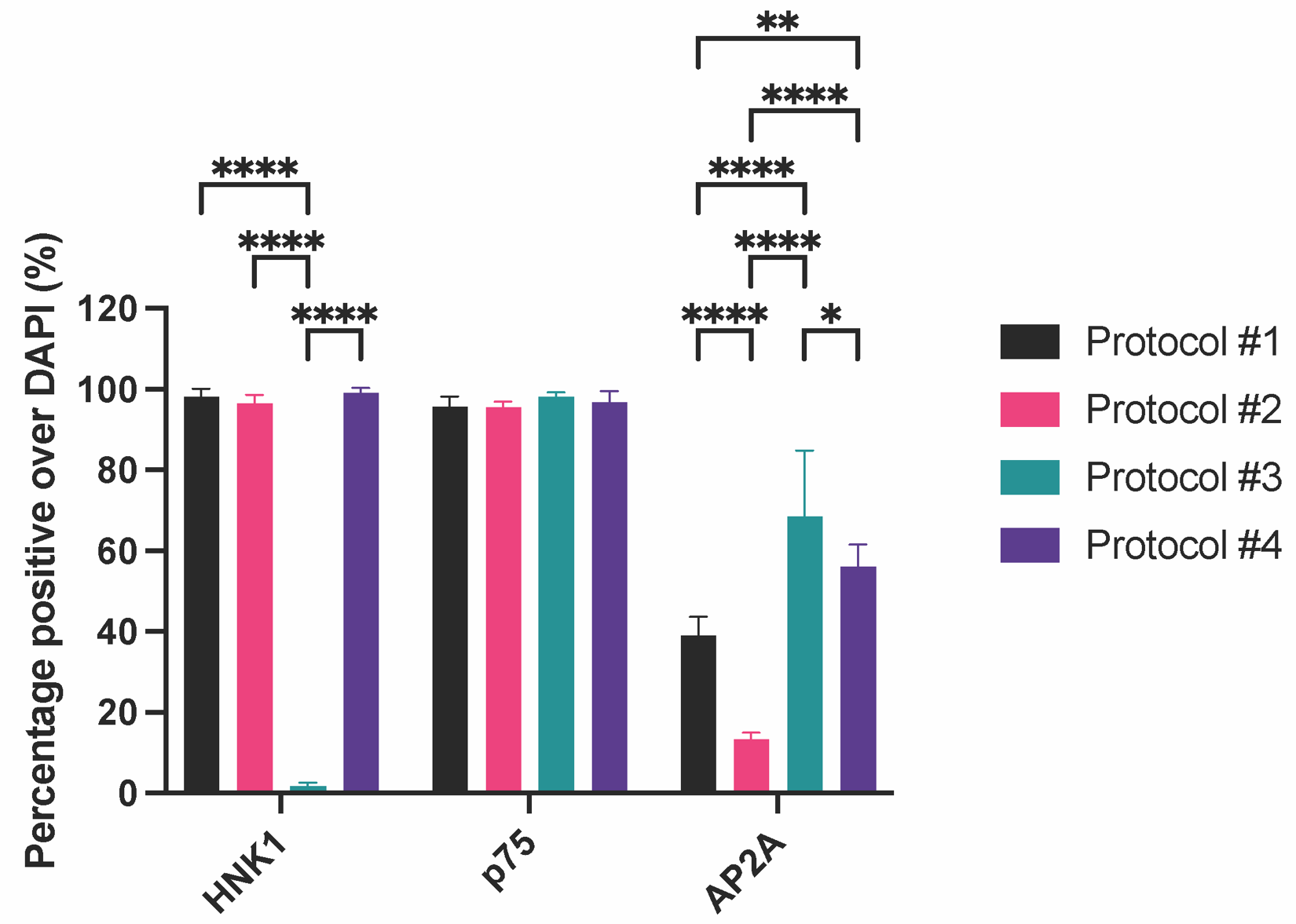


**Supplementary Figure 4: Quantitation of immunofluorescence for NCC markers.** Bar graph shows the percentage of number of positive cells for each protein compared to DAPI stained cells. n=3, error bars represent standard error of mean.


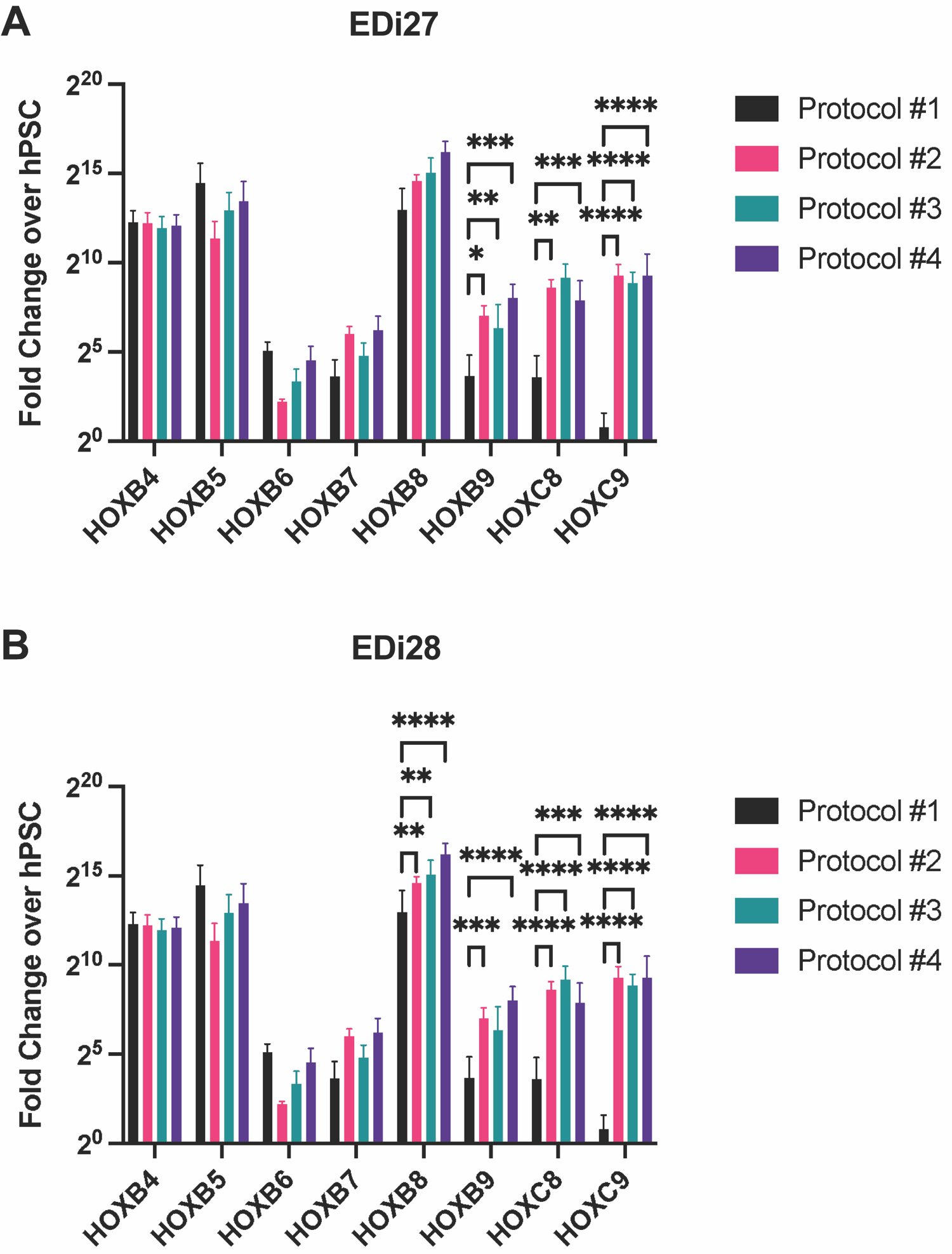


**Supplementary Figure 5: Protocols #2, 3 and 4 generate cells that best resemble tNCC. (A)** EDi27 and **(B)** EDi28 iPSC were differentiated toward tNCC for 8 days and analyzed by RT-qPCR for HOXB4-9 and HOXC8-9. n=3, error bars represent standard error of mean. *p<0.05,**p<0.01,***p<0.001,****p<0.0001.


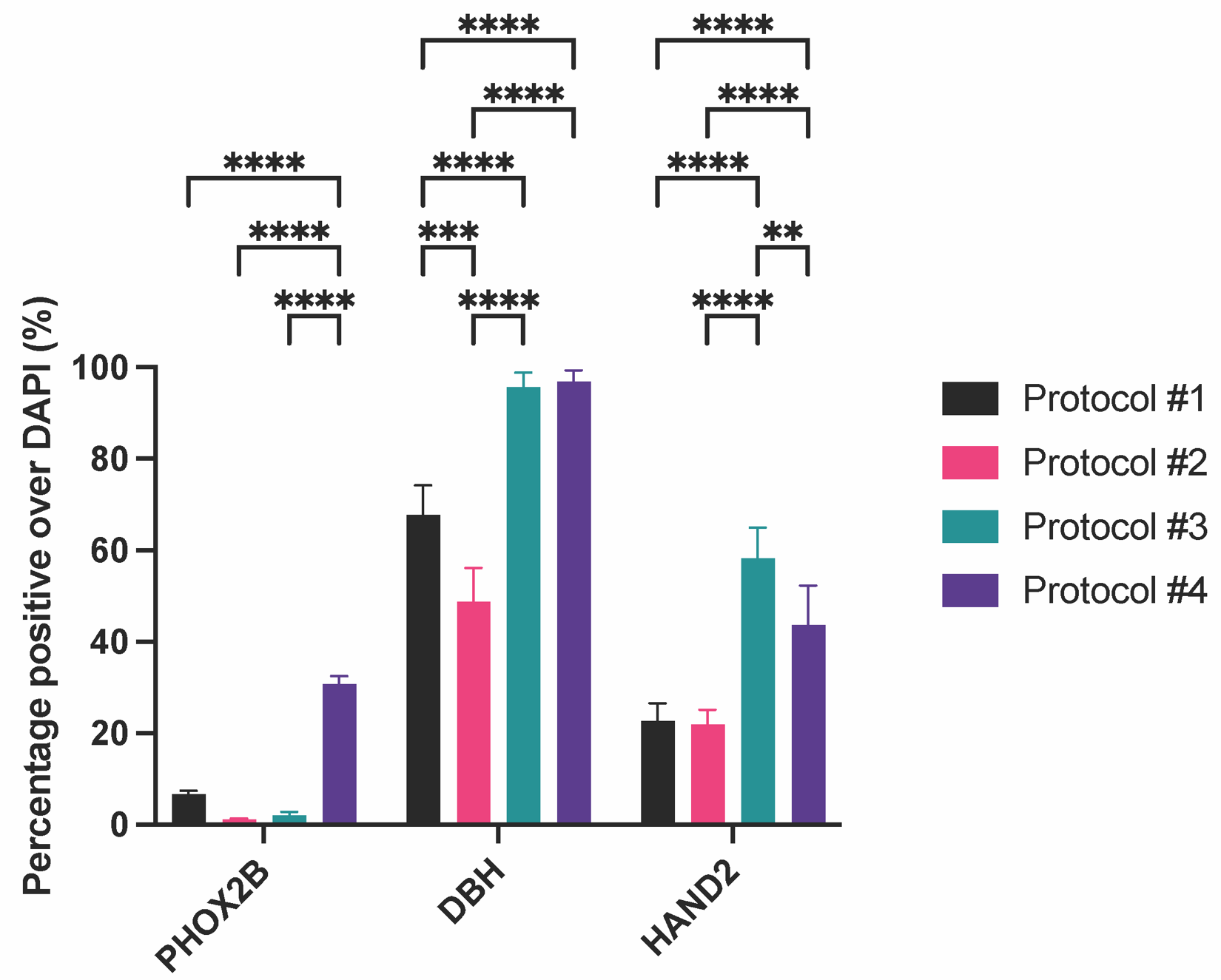


**Supplementary Figure 6: Quantitation of immunofluorescence for SA markers.** Bar graph shows the percentage of number of positive cells for each protein compared to DAPI stained cells. n=3, error bars represent standard error of mean.


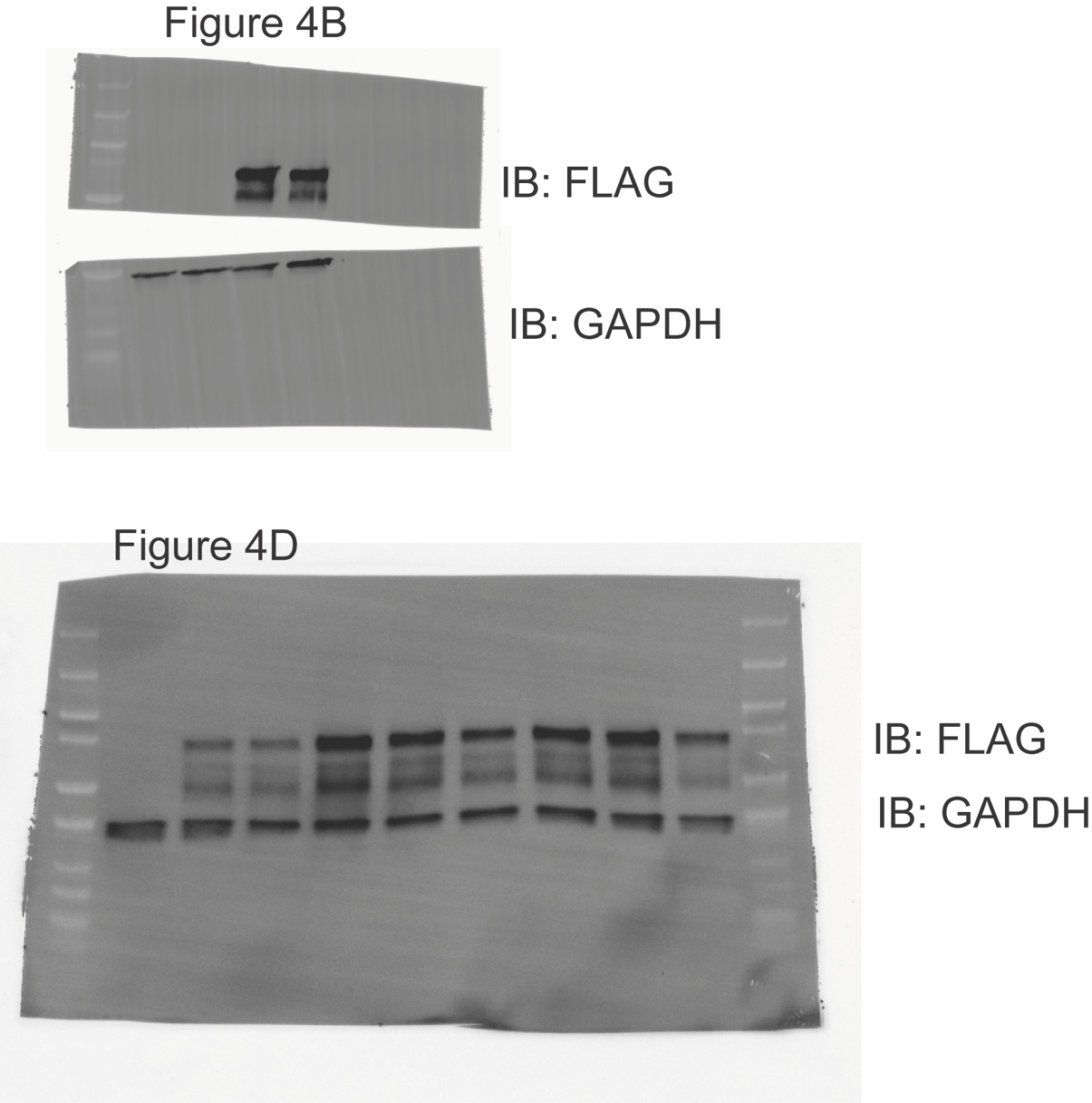


**Supplementary Figure 7:** Uncropped versions of the western blots used in **Figure 4B, D**.


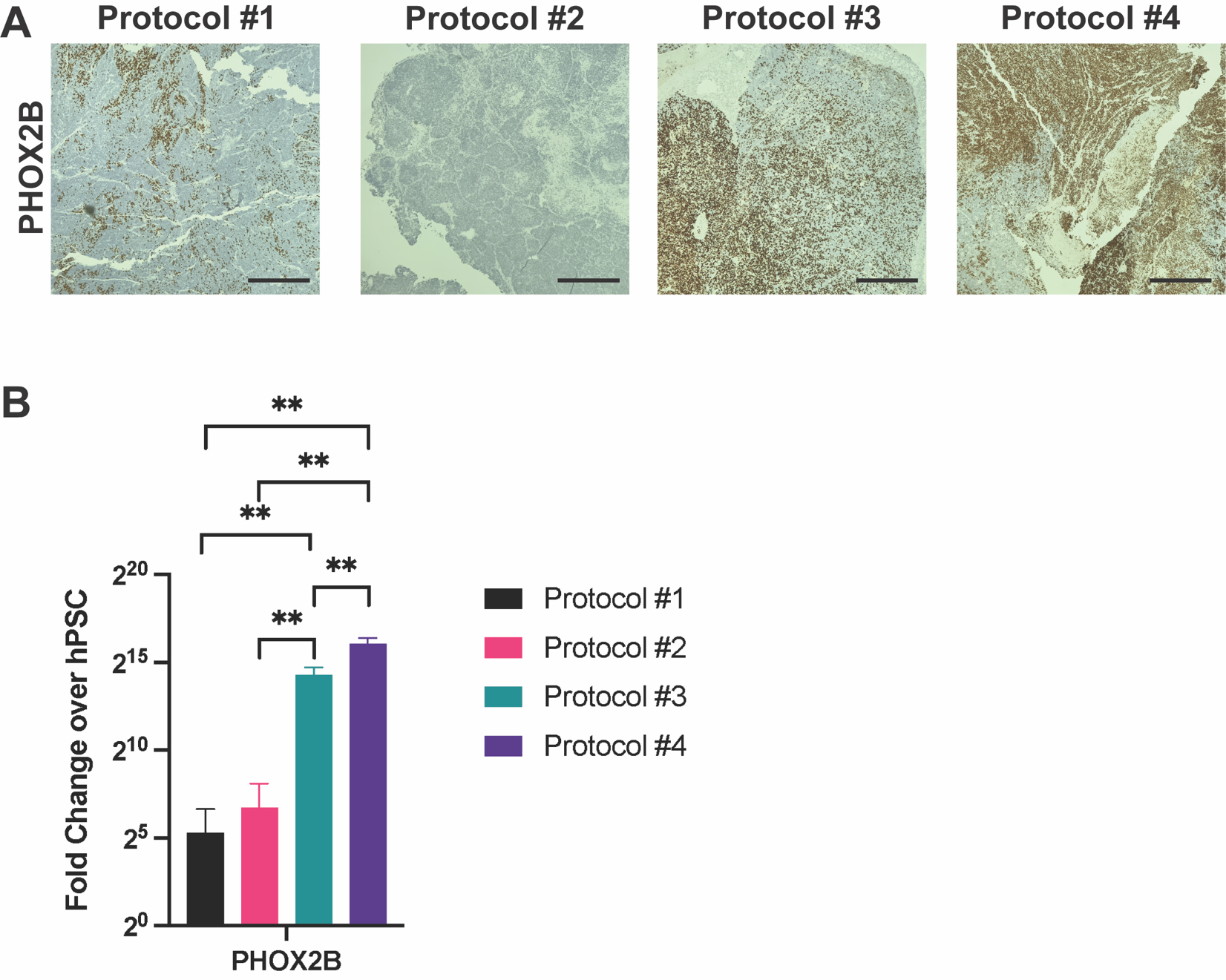


**Supplementary Figure 8: PHOX2B expression in iPSC derived tumors. (A)** Low magnification of IHC for PHOX2B in EDi27 tumors. Scale bars = 560um. **(B)** RT-qPCR analysis of *PHOX2B* in EDi27 tumors. n=3, error bars represent standard error of mean. **p<0.01

**Supplementary Table 1. Differentially expressed genes in Protocol #4 tumors compared to TARGET *MYCN*-amplified neuroblastoma patient samples**
